# Life within a limited radius: Investigating activity space in women with a history of child abuse using global positioning system tracking

**DOI:** 10.1371/journal.pone.0232666

**Published:** 2020-05-11

**Authors:** Franziska Friedmann, Philip Santangelo, Ulrich Ebner-Priemer, Holger Hill, Andreas B. Neubauer, Sophie Rausch, Regina Steil, Meike Müller-Engelmann, Nikolaus Kleindienst, Martin Bohus, Thomas Fydrich, Kathlen Priebe

**Affiliations:** 1 Humboldt-Universität zu Berlin, Berlin, Germany; 2 Karlsruhe Institute of Technology (KIT), Karlsruhe, Germany; 3 DIPF | Leibniz Institute for Research and Information in Education, Frankfurt, Germany; 4 Institute of Psychiatric and Psychosomatic Psychotherapy, Central Institute of Mental Health, Mannheim, Heidelberg University, Heidelberg, Germany; 5 Goethe University Frankfurt, Frankfurt, Germany; 6 McLean Hospital, Harvard Medical School, Boston, Massachusetts, United States of America; 7 Department of Psychiatry and Psychotherapy, Charité –Universitätsmedizin Berlin, Berlin, Germany; Toyama Daigaku, JAPAN

## Abstract

Early experiences of childhood sexual or physical abuse are often associated with functional impairments, reduced well-being and interpersonal problems in adulthood. Prior studies have addressed whether the traumatic experience itself or adult psychopathology is linked to these limitations. To approach this question, individuals with posttraumatic stress disorder (PTSD) and healthy individuals with and without a history of child abuse were investigated. We used global positioning system (GPS) tracking to study temporal and spatial limitations in the participants’ real-life activity space over the course of one week. The sample consisted of 228 female participants: 150 women with PTSD and emotional instability with a history of child abuse, 35 mentally healthy women with a history of child abuse (healthy trauma controls, HTC) and 43 mentally healthy women without any traumatic experiences in their past (healthy controls, HC). Both traumatized groups—i.e. the PTSD and the HTC group—had smaller movement radii than the HC group on the weekends, but neither spent significantly less time away from home than HC. Some differences between PTSD and HC in movement radius seem to be related to correlates of PTSD psychopathology, like depression and physical health. Yet group differences between HTC and HC in movement radius remained even when contextual and individual health variables were included in the model, indicating specific effects of traumatic experiences on activity space. Experiences of child abuse could limit activity space later in life, regardless of whether PTSD develops.

## Introduction

Early traumatic experiences of physical or sexual abuse have a high pathogenicity and are closely associated with increased odds of psychopathology in adulthood [1–3]. Major depressive disorder, anxiety disorders, posttraumatic stress disorder (PTSD) and substance use disorders are major sequelae [1–5]. While the average conditional probability of developing PTSD after any potentially traumatic event ranges between 4.0% and 12.0% [6,7], the conditional probability after experiences of child abuse (CA) is about 35% [2,7–10].

Beyond that, CA is associated with impairments in psychological well-being and general psychosocial functioning in adulthood. Impairments range from decreased life satisfaction, disturbed sexual functioning, relationship difficulties and social withdrawal behavior [11–16] to employment problems and heightened rates of welfare dependence [1,17–20]. Cloitre and colleagues [21] found that in adults with experiences of childhood sexual abuse, emotion regulation and interpersonal problems affected functional impairment to the same degree as PTSD symptom severity, suggesting that impairment in these patients could be affected by factors beyond the PTSD diagnosis. These findings also raise the question whether functional impairments in adult survivors of CA are always associated with PTSD psychopathology and thus only occur in patients with PTSD.

Lately, more studies have incorporated groups of healthy participants with a history of child abuse or participants with subsyndromal PTSD to gain further understanding about the degree of impairments in trauma survivors who do not meet (full) PTSD criteria [7,22–24]. Research from this field contributes to a better understanding of the influence of trauma versus the influence of PTSD psychopathology on functional impairments in traumatized individuals. To date, there are mixed data on functional impairments in traumatized individuals without a psychiatric diagnosis. Some studies show that a history of traumatic events is associated with a variety of clinically relevant difficulties in social functioning, even if full PTSD criteria are not met [7]. Yet other authors report that traumatized individuals without PTSD are less impaired than patients with PTSD and have a similar level of general functioning and quality of life as individuals without any traumatic experiences [23,24], indicating that functional impairments might be connected to psychopathology rather than being related to trauma exposure per se.

The size of one’s activity space beyond the home predominantly serves as an indicator of mobility and is linked to well-being and quality of life [25,26]. Thus, it could serve as a useful indicator of psychosocial functioning in clinical research. Global positioning system (GPS) technology in smartphones can be used as an unobtrusive and cost efficient tool to objectively and continuously characterize activity space through geographic range. Despite the general rise of ambulatory assessment methods (also: experience sampling methods or ecological momentary assessment) as the gold standard for real-time monitoring of dynamic behaviors and symptoms in clinical research and mobile health [27–32], GPS based location information is still an underused data source [33] and little is known about activity space in clinical samples.

So far, GPS technology has been applied in clinical studies with samples including patients with agoraphobia, alcohol dependence and bipolar disorder [34–36]. To our knowledge, no study has used GPS technology to examine activity space in the everyday lives of individuals with full or subsyndromal PTSD. In two studies on college students, higher levels of social anxiety and negative affect were associated with more time and greater likelihood of spending time at home and an increased likelihood of avoiding public areas [37,38]. Participants also showed a reduced likelihood of engaging in leisure activities during evenings and weekends.

In PTSD, activity space might be limited through symptoms like avoidance of fearful situations; negative expectations of others, the world and one’s own safety; hypervigilance; loss of interest; or feelings of alienation—all of which are frequent responses to a trauma and often contribute to maintenance of the disorder [39–42]. Sensitized risk perception and overestimation of dangers and threats [43] could also be relevant limiting factors. Beyond that, co-occurring anxiety disorders or mood disorders might also have an impact on activity space, as higher levels of depression seem to be associated with limited global functioning in individuals with PTSD [44]. Impairments in physical health, which are highly common in PTSD [45], might also limit the mobility and thus the activity space in patients with PTSD.

### The present study

In the present study, activity space and mobility in everyday life were assessed for 7 days via GPS monitoring. We used GPS technology to investigate functional impairment in the sense of a spatially and temporally limited activity space. A group of women with PTSD and emotional instability after child abuse (PTSD group) was compared to two non-clinical control groups of mentally healthy women with and without experiences of child abuse (healthy trauma controls, HTC; healthy controls, HC).

We hypothesized that the PTSD group would show a temporally (less time spent away from home; hypothesis 1a) and spatially (smaller absolute movement radius around their home; hypothesis 1b) reduced activity space compared to the group of HC. As research on long-term trauma effects and functional impairments in subthreshold PTSD is mixed, we also investigated whether the HTC group’s pattern of activity space was more similar to the PTSD group or to the HC group. It was further expected that differences would be moderated by day of the week (weekend days Saturday and Sunday vs. weekdays Monday through Friday; hypothesis 2). This moderation hypothesis is based on the expectation that differences in activity space are more pronounced when individuals have higher degrees of freedom with regard to where they can spend their time. Hence, group differences should be larger on weekend days (vs. weekdays). The influence of potentially confounding context variables (employment, living situation, hometown population) as well as individual health variables (health status, severity of depression) was tested in additional sensitivity analyses. The relationships between activity space dimensions (time spent away, radius) and indicators of psychosocial functioning (avoidance behavior, quality of life and social relationships) were tested in the group of patients with PTSD in an exploratory fashion.

## Materials and methods

### Sample

A total of 272 women participated in the present study. The sample consisted of 180 women with PTSD according to DSM-5 after child abuse, defined as sexual or physical abuse before the age of 18; 41 mentally healthy women who had also experienced child abuse (healthy trauma controls, HTC); and 51 mentally healthy women, who had never experienced any traumatic event (healthy controls, HC). Enrollment was restricted to women aged 18–65 years.

The women with PTSD were recruited from a multicenter randomized controlled trial study (Treating Psychosocial and Neural Consequences of Childhood Interpersonal Violence in Adults, German Clinical Trials registration number: DRKS00005578) that compared Dialectical Behavior Therapy for PTSD [46] with Cognitive Processing Therapy [47]. Details of the study design are published elsewhere [48]. Participation in the present study took place during the baseline assessment period of the multicenter study.

Inclusion criteria for the PTSD group were the diagnosis of PTSD related to sexual or physical abuse before the age of 18 and at least three DSM-5 criteria of borderline personality disorder (BPD), including emotional instability (criterion 6). Exclusion criteria were a lifetime diagnosis of schizophrenia or bipolar I disorder, intellectual disability, current substance dependence, body mass index <16.5, current pregnancy, unstable life situation (homelessness or ongoing victimization) or a suicide attempt within the last two months.

HTCs and HCs were recruited through the databases at the Department for Psychosomatic Medicine and Psychotherapy Mannheim and the Humboldt-Universität zu Berlin, as well as through print and web advertisements. Participants received an average expense allowance of 100 EUR for their participation, with variation due to differences in time expenditure for initial diagnostic clarification. As in the patient group, the main inclusion criterion in the HTC group was the experience of child abuse. Exclusion criteria for both control groups (HTC and HC) included a lifetime diagnosis of any DSM-IV Axis I disorder or BPD, the intake of psychotropic drugs, any psychotherapeutic treatment in the past or present and intellectual disability.

Of the 272 participants, 44 were excluded from data analysis because of entirely missing (21 PTSD, 4 HTC, 5 HC) or insufficient (<2 days of GPS data: 9 PTSD, 2 HTC, 3 HC) GPS data due to technical problems. This yielded a final sample of 228 participants (150 PTSD, 35 HTC, 43 HC). Eighteen of the 35 HTCs were recruited from a list of participants involved in a prior study 24]. The three groups were matched for age (PTSD: 35.5 (*SD* = 10.9); HTC: 32.1 (*SD* = 12.3); HC: 32.3 (*SD* = 11.5); *F*[2,223] = 2.15, *p* = .119). Written informed consent was obtained from all participants, and the ethics committees of all three involved centers (Ethik-Kommission II of the Medical Faculty of the Central Institute of Mental Health, Mannheim and Heidelberg University; Ethikkomission of the Psychologisches Institut of the Humboldt-Universität zu Berlin; Ethikkommission of the Fachbereich für Psychologie und Sportwissenschaften of the Goethe University Frankfurt) approved the study (reference number: 2013-635N-MA).

### Procedure

All participants were invited to diagnostic sessions to assess the inclusion- and exclusion criteria. Trained psychologists conducted the Structured Clinical Interview for DSM-IV (SCID) [49], the Global Assessment of Functioning Scale (GAF) [50], and the BPD section of the International Personality Disorder Examination (IPDE) [51]. An intelligence test (Mehrfachwahl-Wortschatz-Intelligenztest) [52] was obtained to rule out intellectual disability. The assessment of general psychopathology comprised the Brief Symptom Inventory (BSI) [53] and the Beck Depression Inventory-II (BDI-II) [54]. Quality of life and its four main domains (physical health, psychological health, social relationships and environment) were assessed through the WHOQOL-BREF [55], subjective health via EQ-5D-3L [56]. In the group of patients with PTSD, the Clinician Administered PTSD Scale for DSM-5 (CAPS-5) [57] and the Life Events Checklist for DSM-5 (LEC-5) [58] were applied for PTSD assessment. PTSD severity and trauma history were assessed via the PTSD Checklist for DSM-5 (PCL-5) [59] in the PTSD and HTC groups and the Childhood Trauma Questionnaire (CTQ) [60] in all groups. All participants received a smartphone, which recorded their movement pattern for seven days via an application [61]. Participants were instructed to take the smartphone wherever they went.

To save battery power, the app used an energy-optimizing algorithm [62]. Coordinates were measured in five-second intervals, but only when the acceleration sensor of the smartphone detected that the smartphone was moved. Measuring location via GPS has a high accuracy (+/-10 m), but it requires substantial battery power and the signal cannot be recorded sufficiently everywhere (e.g. indoor, in trains etc.). Therefore, the app preferentially measured location based on WiFi-routers (accuracy +/-40 m) and cell tower coordinates (accuracy +/-200-3000 m) and switched to GPS only if the location measure was insufficient. The app stored the time, coordinates (latitude and longitude), and accuracy of location measurements.

### Data preprocessing

Data (time spent away from home per day, maximum radius around home per day) were analyzed using a Matlab (version 8.5.1) script. Distance was computed based on the spatial difference between two subsequent measures of latitude and longitude. First, the difference (in degrees) was computed for latitude and longitude separately. Then the degree differences were recalculated as differences in km, correcting for the fact that the distances between two lines of longitude are largest at the equator and zero at the poles. Third, the distance (vector) between two coordinates was computed using Pythagoras' theorem. Fourth, all measured samples from a given day were added. Due to inaccuracies in the location measures, samples were only included when accuracy was better than +/-100 m and movement speed was below 400 km/h (i.e. the fastest German trains—very high artifactual speed values can occur when, for example, a WiFi router is moved away from the position where it was registered, such as mobile routers mounted in trains or trucks). Home was conservatively defined as a radius of 500 m around the geo code the participants logged as their home location. Visual inspection of the data showed that distance (absolute movement radius) and time measures (minutes away from home) were strongly positively skewed; these values were therefore log transformed prior to the analyses (log_dist = log(dist + 1); log_time = log(time + 1); where log is the natural logarithm).

### Statistical analysis

All statistical analyses were performed using R version 3.5.1 for Windows. A significance level of .05 (two-tailed) was applied for all analyses. To test our research hypotheses, we employed linear mixed-models to account for the nested data structure (with repeated measurements on the within-person level [Level 1] nested within participants [between-person level; Level2]). We set up a model predicting (logarithmized) time away from home (H1a) or (logarithmized) movement radius (H1b) from group (PTSD vs. HTC vs. HC) and day of the week (H2, variable *weekend* coded 0 for weekdays and 1 for weekend days). For both dependent variables, an empty two-level model (without predictors) was set up first (Model 0a for the dependent variable time away from home and Model 0b for movement radius). Next, we added main effects of group and *weekend* as predictors: Specifically, the variable group was entered with two dummy coded variables, comparing the HC group to the HTC and the HC group to the PTSD group; hence, the HC group served as the reference group. In these models, the dependent variable Y (logarithmized time away from home [Model 1a] or logarithmized movement radius [Model 1b]) for individual *i* on day *t* was predicted as follows:

Level 1:

$$Y_{it}=\beta_{0i}+\beta_{1i}∙{weekend}_{it}+\varepsilon_{it}$$ (1)

Level 2:

$$\beta_{0i}=\gamma_{00}+\gamma_{01}∙{PTSD}_{i}+\gamma_{02}∙{HTC}_{i}+\upsilon_{0i}$$ (2)

$$\beta_{1i}=\gamma_{10}+\upsilon_{1i}$$ (3)

The *γ* parameters represent the fixed effects, the υ parameters the random effects. The parameter *β*_0*i*_ is individual *i*'s intercept, *β*_1*i*_ is this individual’s estimated difference in Y between weekend days and weekdays. Person *i*'s intercept (*β*_0*i*_) is predicted by the two dummy variables comparing the PTSD group to the HC group and the HTC group to the HC group, respectively. These dummy variables are between-person (Level 2) predictors and therefore represented in the first Level 2 equation (Eq 2). The parameter *γ*_10_ (Eq 3) is the estimated average difference in the dependent variable on the weekend versus weekdays. Correspondingly, the parameter *γ*_00_ is the estimated average value on the dependent variable (logarithmized time away from home or logarithmized movement radius) in the HC group during the week. *γ*_01_ is the estimated difference between the average value in the dependent variable between the HC group and the PTSD group, and *γ*_02_ is the estimated difference between the HC group and the HTC group. The Level-2 residuals (υ_0*i*_ and υ_1*i*_) capture inter-individual differences in the intercept and the difference in Y between weekend days and weekdays. Finally, *ε_it_* is a day- and person-specific residual. To implement the moderation hypothesis 2, we added two-way interactions of the group indicators variable with *weekend* (Models 2a and 2b):

Level 1:

$$Y_{it}=\beta_{0i}+\beta_{1i}∙{weekend}_{it}+\varepsilon_{it}$$ (4)

Level 2:

$$\beta_{0i}=\gamma_{00}+\gamma_{01}∙{PTSD}_{i}+\gamma_{02}∙{HTC}_{i}+\upsilon_{0i}$$ (5)

$$\beta_{1i}=\gamma_{10}+\gamma_{11}∙{PTSD}_{i}+\gamma_{12}∙{HTC}_{i}+\upsilon_{1i}$$ (6)

Note that Eqs (4) and (5) are equivalent to Eqs (1) and (2), respectively. Here, *γ*_11_ estimates the difference between the HC and PTSD group in the effect of the predictor *weekend* (i.e., the difference between these two groups in the difference between weekend days and weekdays). Correspondingly, *γ*_12_ estimates the difference between the HC and HTC group in the effect of the predictor *weekend*.

To compare the HTC to the PTSD group, we repeated these models using two different dummies (PTSD vs. HTC and PTSD vs. HC). Only the effects involving the former contrast will be reported as exploratory analyses. For all models, an unstructured Level 2 random effect matrix was estimated (i.e. the random effects υ_0*i*_ and υ_1*i*_ were allowed to covary). Significance of the fixed effects was determined using the Kenward-Roger approximation and restricted maximum likelihood estimation (REML) as implemented in the lmerTest package [63].

To examine the robustness of our findings, we performed additional sensitivity analyses in which we first controlled for context variables: employment status (dichotomous: 0 = unemployed, 1 = employed), living situation (dichotomous: 0 = living alone, 1 = living with other people), and hometown population (number of inhabitants of one’s hometown). Next, we added level of depression (BDI-II) and health status (EQ-5D), to examine if findings remain robust after controlling for individual health variables. Detailed results of these models are reported in the supplemental online material (S1 Table and S2 Table).

We further examined the correlations of mean (logarithmized) time away and mean (logarithmized) movement radius with indicators of psychosocial functioning (experiential avoidance, quality of life, and social relationships) in the PTSD group only.

## Results

### Sample characteristics

The demographic and clinical characteristics of all three groups are provided in Table 1. The chi-square test revealed no statistically meaningful group differences in educational level distribution (χ^2^[6] = 13.50, *p* = .197) or employment status (χ^2^[2] = 5.65, *p* = .059). One-way analyses of variance revealed significant group differences regarding clinical characteristics, with individuals with PTSD showing significantly lower levels of functioning and health and higher levels of psychological distress and depressive symptoms (GAF: *F*[2,224] = 701.44, *p* < .001; EQ-5D health status: *F*[2,211] = 105.78, *p* < .001; BSI: *F*[2,221] = 221.57, *p* < .001; BDI: *F*[2,223] = 290.97, *p* < .001). Significant differences were also found regarding the distribution of index trauma, with a higher proportion of sexual abuse in the PTSD group compared to the HTC (χ^2^[1] = 9.73, *p* = .002). Per definition the HC and the HTC did not suffer from any mental disorder, whereas the participants suffering from PTSD had a mean of three co-occurring Axis I disorders, with anxiety disorders (60%), and mood disorders (67%) being most frequent. About half of the patients with PTSD (52%) had a comorbid borderline personality disorder.

**Table 1:** Demographic and clinical variables of the PTSD group, healthy trauma controls and healthy controls.

	PTSD (N = 150)	HTC (N = 35)	HC (N = 43)	Difference test
Age (years), M ± SD	35.5 ± 10.9	32.1 ± 12.3	32.3 ± 11.5	F(2,223) = 2.15, p = .119
Education (years)				
9 years or fewer, N (%)	31 (20.9)	2 (6.1)	4 (9.5)	χ2 (6) = 13.50, p = .197
10 years, N (%)	51 (34.5)	9 (27.3)	13 (31.0)	
12 years, N (%)	57 (38.5)	21 (63.6)	24 (57.1)	
Other, N (%)	9 (6.1)	1 (3.0)	1 (2.4)	
Employment				
Employed, N (%)	74 (49.3)	25 (71.4)	24 (55.8)	χ2 (2) = 5.65, p = .059
Hours per week, M ± SD	11.8 ± 16.8	15.5 ± 15.0	11.3 ± 15.3	F(2,211) = .78, p = .462
Living situation				
Alone, N (%)	45 (30.0)	17 (48.6)	8 (19.0)	χ2 (12) = 21.11, p = .174
Shared apartment, N (%)	17 (11.3)	3 (8.6)	10 (23.8)	
With parents, N (%)	8 (5.3)	1 (2.9)	4 (9.5)	
With partner, N (%)	45 (30.0)	11 (31.4)	16 (38.1)	
With children, N (%)	16 (10.7)	1 (2.9)	2 (4.8)	
With partner and children, N (%)	13 (8.7)	2 (5.7)	1 (2.4)	
Other, N (%)	6 (4.0)	0 (0.0)	1 (2.4)	
Hometown population, M ± SD	1007300	1679084	2051835	F(2,224) = 9.28, p < .001
	± 1411951	± 1699108	± 1651647	
Psychosocial functioning				
Level of functioning (GAF), M ± SD	49.6 ± 8.3	88.9 ± 7.5	91.4 ± 5.5	F(2,224) = 701.44, p < .001
Psychological distress (BSI), M ± SD	1.8 ± 0.6	0.2 ± 0.2	0.2 ± 0.2	F(2,221) = 221.57, p < .001
Depression severity (BDI-II), M ± SD	34.1 ± 11.0	3.0 ± 4.0	3.3 ± 3.1	F(2,223) = 290.97, p < .001
Health status (EQ-5D), M ± SD	48.1 ± 20.6	82.1 ± 13.6	87.3 ± 9.4	F(2,211) = 105.78, p < .001
WHOQOL-BREF-global score, M ± SD	45.8 ± 10.0	75.9 ± 11.0	90.3 ± 14.3	F(2,219) = 310.33, p < .001
WHOQOL-social relationships, M ± SD	18.8 ± 17.3	49.0 ± 17.1	60.7 ± 17.7	F(2,218) = 115.93, p < .001
Comorbidities				
Any anxiety disorder (SCID), N (%)	83 (59.7)	0	0	
Any mood disorder (SCID), N (%)	89 (66.9)	0	0	
Any other disorder (SCID), N (%)	36 (34.9)	0	0	
Comorbid Axis I disorders (SCID), M ± SD	3.0 ± 1.5	0	0	
BPD diagnosis (IPDE), N (%)	77 (51.3)	0	0	
Index trauma				
Sexual abuse, N (%)	113 (75.3)	17 (48.6)	0	χ2 (1) = 9.73, p = .002
Physical abuse only, N (%)	37 (24.7)	18 (51.4)	0	
Age of onset, M ± SD	7.9 ± 4.3	8.5 ± 5.0	0	t(183) = -.73, p = .464
Duration of abuse, M ± SD	6.8 ± 6.1	4.5 ± 5.5	0	t(183) = 2.03, p = .044
PTSD severity and trauma history				
CAPS-total score, M ± SD	40.7 ± 9.7	-	-	
PCL-5-total score, M ± SD	49.3 ± 11.3	4.5 ± 4.9	-	t(175) = 20.67, p < .001
PCL-5-avoidance of reminders, M ± SD	2.7 ± 1.2	0.3 ± 0.6	-	t(182) = 11.21, p < .001
CTQ-total score, M ± SD	82.4 ± 18.0	59.3 ± 15.9	43.6 ± 5.0	F(2,221) = 107.41, p < .001
CTQ-sexual abuse, M ± SD	14.9 ± 7.1	9.6 ± 6.4	5.1 ± 0.6	F(2,221) = 43.49, p < .001
CTQ-physical abuse, M ± SD	12.6 ± 6.0	9.3 ± 4.5	5.2 ± 0.5	F(2,221) = 33.85, p < .001
CTQ-emotional abuse, M ± SD	15.0 ± 4.2	13.0 ± 6.1	7.1 ± 2.4	F(2,221) = 55.05, p < .001
CTQ-emotional neglect, M ± SD	19.3 ± 4.6	12.7 ± 5.2	8.4 ± 3.2	F(2,221) = 108.99, p < .001
CTQ-physical neglect, M ± SD	11.9 ± 4.2	7.5 ± 3.2	6.3 ± 1.7	F(2,221) = 47.69, p < .001

*M* = mean; *SD* = standard deviation; *GAF* = Global Assessment of Functioning Scale; *BSI* = Brief Symptom Inventory; *BDI-II* = Beck Depression Inventory-II; *SCID* = Structured Clinical Interview for DSM-IV; *IPDE* = International Personality Disorder Examination; *CAPS* = Clinician Administered PTSD Scale for DSM-5; *PCL-5* = PTSD Checklist for DSM-5; *CTQ* = Childhood Trauma Questionnaire; *WHOQOL-BREF* = World Health Organization Quality of Life-BREF. Dashes indicate that data were not obtained.

#### Time away from home

**Fig 1:**
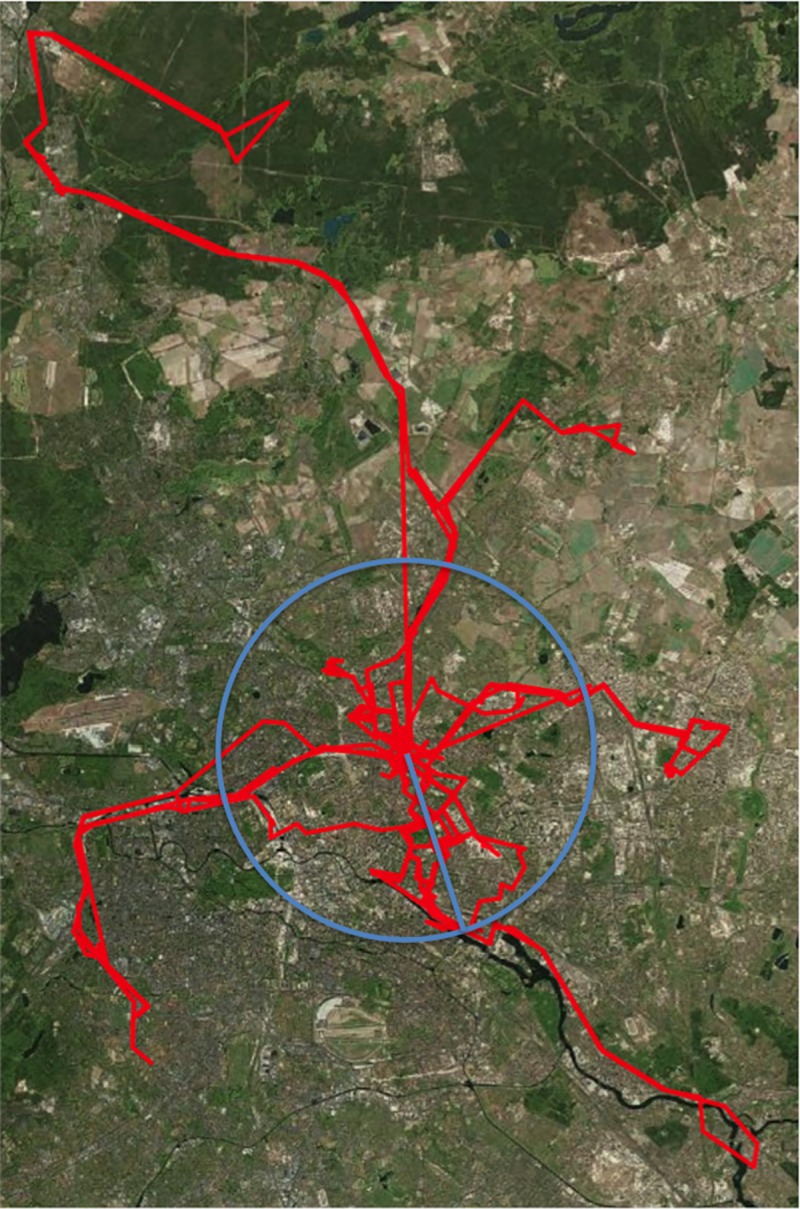
Example of seven-day GPS activity pattern of a patient with PTSD.

Results of the multilevel models predicting (logarithmized) time away from home can be found in Table 2. Fig 1 depicts the activity pattern of a single patient with PTSD from the study center over the course of one week. In contrast to hypothesis 1a, there was no statistically significant difference in time away from home between the HC group and either the PTSD group, *b* = -0.383, *p* = .228, or the HTC group, *b* = -0.644, *p* = .124 (see Model 1a). There was a main effect of the predictor *weekend*, *b* = -0.906, *p* < .001, indicating that across all groups participants spent less time away from home on the weekend vs. weekdays. Adding interactions of group with *weekend* did not improve model fit, χ^2^(2) = 1.729, *p* = .421, and none of the interaction effects was statistically significant, *p* > .193 for all.

**Table 2:** Multilevel models predicting (logarithmized) time away from home.

	Model 0a	Model 1a	Model 2a
	Fixed effects		
Intercept	3.657*** (0.121)	4.271*** (0.281)	4.194*** (0.286)
PTSD	-	-0.383 (0.317)	-0.287 (0.324)
HTC	-	-0.644 (0.417)	-0.560 (0.426)
HC	-	(Reference)	(Reference)
Weekend^a^	-	-0.906*** (0.123)	-0.573* (0.285)
PTSD x Weekend^a^	-	-	-0.421 (0.323)
HTC x Weekend^a^	-	-	-0.367 (0.424)
HC x Weekend^a^	-	-	(Reference)
	Random effects (variances)		
Intercept	2.732	2.872	2.872
Weekend^a^	-	1.304	1.307
Residual (Level 1)	3.540	3.037	3.037

Table depicts unstandardized coefficients (standard errors in parentheses). Time away from home was logarithmized prior to the analyses. For a formal description of Model 1a please see Eqs (1)–(3); Model 2a corresponds to Eqs (4)–(6). Number of observations = 1,563; number of participants = 228.

*p < .05

**p < .01

***p < .001

^a^: weekday = 0, weekend = 1.

The activity pattern of a patient with PTSD (participant ID 311625) from the study center Berlin over the course of one week. The circle with the line illustrates the movement radius of one specific study day during that week. Aerial imagery from ESRI/ArcGIS.

**Fig 2:**
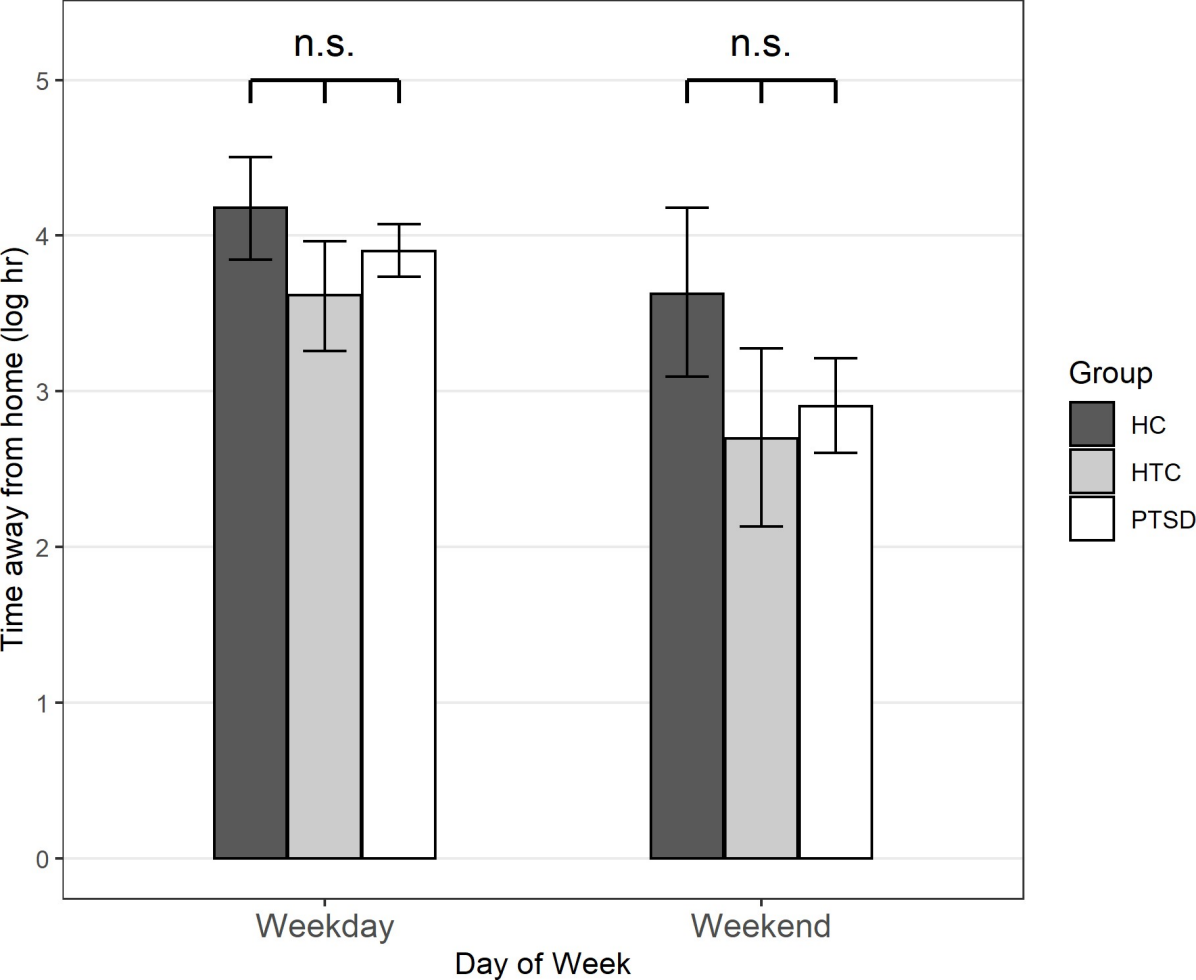
Mean (logarithmized) time away from home on weekdays and weekend days for all groups. Mean (logarithmized) time away from home on weekdays (left) and weekend days (right), separately for the healthy control group (HC; dark grey), the healthy trauma control group (HTC; light grey), and the patient group (PTSD; white). Statistical significance in the figure refers to the results from the main analyses (no covariates): *p < .05, n.s.: *p* > .05.

Exploratory analyses revealed no statistically significant difference between the PTSD group and the HTC group, *b* = -0.261, *p* = .449. The difference between the two groups was also not moderated by day of the week, *b* = 0.054, *p* = .878. Fig 2 depicts mean levels in (logarithmized) time away from home separately for the three groups and for days during the week vs. on the weekends.

We next added employment status, living situation and hometown population as covariates (see Model 3a in S1 Table). Findings using the primary contrasts (HC vs. PTSD and HC vs. PTSD) showed that the group differences and the group x *weekend* interactions remained statistically non-significant, *p* > .234 for all. Further exploratory analyses showed that after controlling for these three covariates, the difference in time away from home between the PTSD group and the HTC group was statistically significant, *b* = - .690, *p* = .045, with HTC participants spending less time away from home compared to participants with PTSD. Finally, we entered depression and health status as additional covariates (see Model 4a in S1 Table). There was no effect of these two predictors on time away from home, *p* > .275 for all. Including these covariates did not change the pattern of results compared to the previous model: the difference between the HC group and the HTC group was not statistically significant, *b* = -311, *p* = .468, and neither was the differences between the HC group and the PTSD group, *b* = -.823, *p* = .135. The difference between the PTSD group and the HTC group remained statistically significant, *b* = -1.134, *p* = .043.

#### Absolute movement radius

In line with research hypothesis 1b, there was a main effect for the contrast comparing the HC group to the PTSD group, *b* = -0.497, *p* = .003, suggesting that absolute movement radius was larger for the HC group than the PTSD group (see Table 3, Model 1b). There was not a statistically significant difference in movement radius between the HC group and the HTC group, *b* = -0.405, *p* = .061. Results further yielded a statistically significant main effect of the predictor *weekend*, *b* = -0.312, *p* < .001, suggesting that absolute movement radius was larger on weekdays than on the weekend.

**Table 3:** Multilevel models predicting (logarithmized) movement radius.

	Model 0b	Model 1b	Model 2b
	Fixed effects		
Intercept	1.645*** (0.064)	2.126*** (0.146)	2.002*** (0.151)
PTSD	-	-0.497** (0.164)	-0.344* (0.171)
HTC	-	-0.405 (0.215)	-0.258 (0.225)
HC	-	(Reference)	(Reference)
Weekend^a^	-	-0.312*** (0.081)	0.186 (0.185)
PTSD x Weekend^a^	-	-	-0.618** (0.210)
HTC x Weekend^a^	-	-	-0.593* (0.276)
HC x Weekend^a^	-	-	(Reference)
	Random effects (variances)		
Intercept	0.719	0.728	0.726
Weekend^a^	-	0.654	0.612
Residual (Level 1)	1.381	1.203	1.202

Table depicts unstandardized coefficients (standard errors in parentheses). Movement radius was logarithmized prior to the analyses. For a formal description of Model 1b please see Eqs (1)–(3); Model 2b corresponds to Eqs (4)–(6). Number of observations = 1,563; number of participants = 228.

*p < .05

**p < .01

***p < .001

^a^: weekday = 0, weekend = 1.

**Fig 3:**
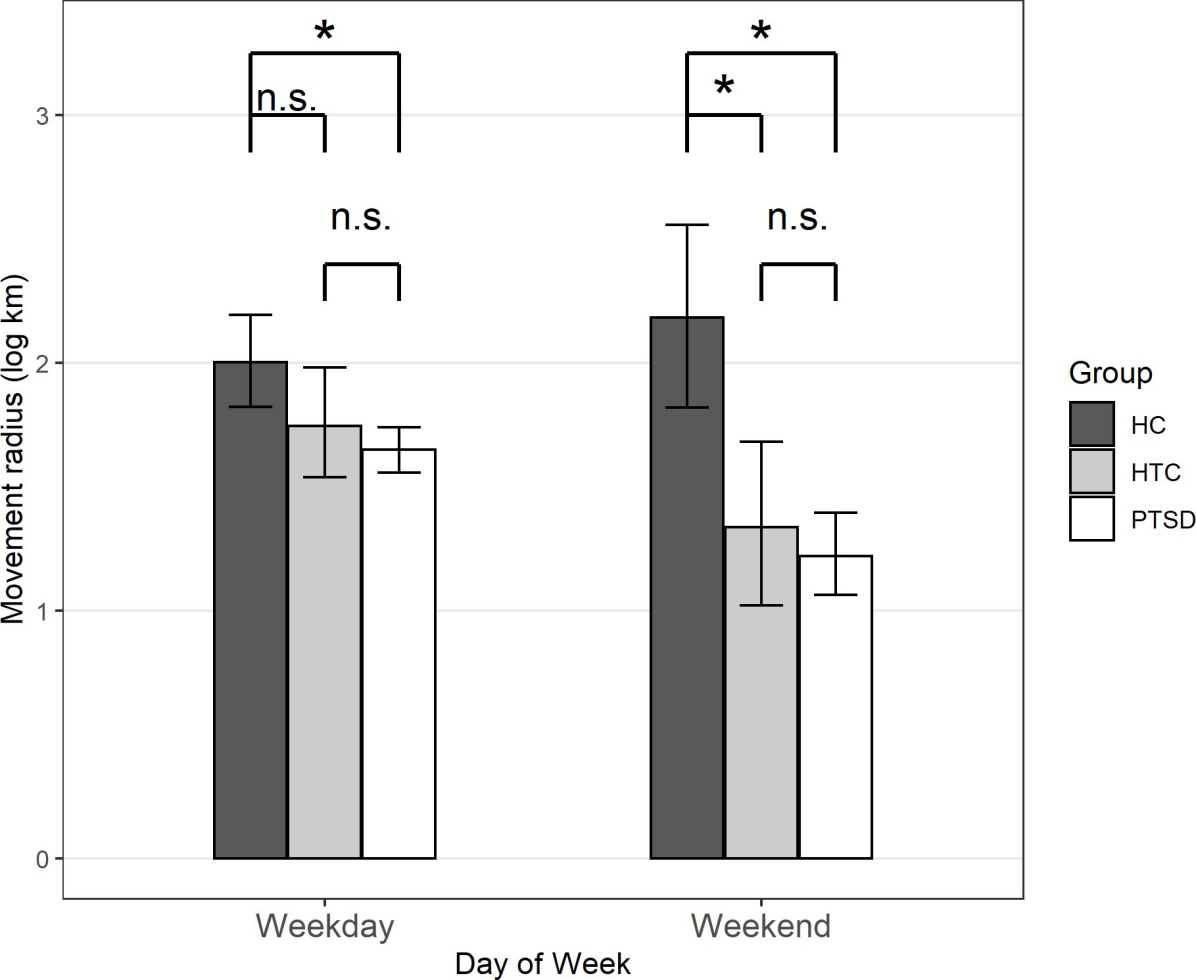
Mean (logarithmized) movement radius on weekdays and weekend days for all groups. Mean (logarithmized) movement radius on weekdays (left) and weekend days (right), separately for the healthy control group (HC; dark grey), the healthy trauma control group (HTC; light grey), and the patient group (PTSD; white). Statistical significance in the figure refers to the results from the main analyses (no covariates): *p < .05, n.s.: *p* > .05.

Adding interaction effects (Model 2b) further showed that, in line with hypothesis 2, the group differences were moderated by day of the week. Specifically, both the difference in movement radius between the HC group and the PTSD group, *b* = -0.618, *p* = .004, and the difference between the HC group and the HTC group, *b* = -0.593, *p* = .032, were larger on the weekends than during the week. Splitting the data into weekend days and weekdays showed that on weekends, movement radius was smaller in both the PTSD group, *b* = -0.967, *p* < .001, and in the HTC group, *b* = -0.849, *p* = .005, compared to the HC group. On weekdays, only the difference between the HC group and the PTSD group, *b* = -0.340, *p* = .048, but not the difference between the HC group and the HTC group, *b* = -0.254, *p* = .261, was statistically significant. There was not a statistically significant difference in average movement radius between the PTSD group and the HTC group in exploratory analyses, *b* = 0.092, *p* = .604, and no significant moderation by day of the week, *b* = 0.024, *p* = .915 (see Fig 3).

Fig 3 depicts mean levels in (logarithmized) movement radius separately for the three groups and for days during the week vs. on the weekends.

When adding the first set of covariates (context variables: employment status, living situation, hometown population), findings remained largely unaltered (see Model 3b in S2 Table): The group x *weekend* interactions remained statistically significant, as did the group differences on the weekends. The difference between the HC group and the PTSD group during the week was, however, no longer statistically significant, *b* = -0.199, *p* = .263. When depression and health status were added additionally (see Model 4b in S2 Table), the group x *weekend* interactions remained statistically significant. None of the three group differences during the week were statistically significant in these models, *p* > .352. On weekends, the difference between the HC and HTC groups remained statistically significant, *b* = -.715, *p* = .027, whereas the difference between the HC and PTSD group did not, *b* = -.003, *p* = .995.

#### Correlations with psychopathology

We examined the associations of average time away from home and movement radius across the whole observation period with avoidance of external reminders (PCL-5 item 7), quality of life (WHOQOL-BREF), and social relationships (WHOQOL-BREF social relationships domain). To that end, we first computed mean (logarithmized) time away and movement radius and computed correlations with the questionnaire measures in the PTSD group. Results (see Table 4) showed that average time away from home was unrelated to any of the questionnaire measures, |*r*| < .04 for all. Movement radius was only related to overall quality of life, *r* = .24. All other correlations failed to reach statistical significance, *p* > .05 for all.

**Table 4:** Correlations of time away from home and movement radius with psychopathology and indicators of psychosocial functioning.

		2	3	4	5
1	Time away	.70**	-.04	.01	.04
2	Movement radius		-.07	.24*	.14
3	Avoidance of reminders			-.16	-.04
4	Quality of life				.61**
5	Social relationships				

Time away and movement radius were logarithmized and then aggregated into a mean. N = 145–150.

* *p* < .01

**p < .001.

## Discussion

We used real time GPS monitoring to assess the movement patterns of female patients with PTSD and healthy women with and without experiences of child abuse. Consistent with our hypotheses, patients with PTSD stayed in a more restricted area around their homes compared to the group of mentally healthy women without any traumatic experiences (HC). Interestingly, HTC did not generally differ from the HC or PTSD group regarding their movement radius. However, on the weekends, both groups of traumatized women (PTSD and HTC) stayed in a smaller movement radius compared to the HC, indicating a potential effect of trauma on activity space for situations when degrees of freedom for individual behavior are higher. Even when context variables like employment, living with others or the size of the hometown population were included in the model, these group differences remained. When controlling for depression and health status, there was no difference between patients with PTSD and HC in their movement radius, yet the difference between HTC and HC remained. Hence, physical and psychological health partially account for the difference between the PTSD group and the HC group, but not for the differences between the HTC group and the HC group. This suggests that while some differences in activity space found between the three groups may be due to correlates of the psychopathology of PTSD, there seem to be specific effects of trauma experiences on activity space above and beyond these effects when individuals’ degrees of freedom for their activities is high (e.g., on the weekends).

The findings in the GPS measures are partially in line with some previous studies showing reduced general functioning and increased avoidance behavior and interpersonal problems in adulthood after child abuse [1,17–20]. At the same time, the finding that HTC were more similar to HC in self-reported measures of psychosocial functioning was consistent with studies concluding that healthy trauma controls show high levels of self-reported psychosocial functioning [23–24].

Contrary to our hypotheses, patients with PTSD did not spend significantly less time away from home compared to HC. This might indicate that after experiences of highly traumatic events such as sexual or physical abuse, spending time outside is not per se evaluated as being dangerous, but moving further away from a well-known area around one’s home might induce feelings of insecurity more than it does in women without a history of traumatic experiences. Possible explanations might be a heightened risk perception or a lack of trust in unfamiliar situations. Unlike radius, which was significantly related to quality of life in the PTSD group, time spent away from home was not significantly related to quality of life and might accordingly be a weaker indicator of psychosocial functioning.

There are several studies showing that the experience of a traumatic event results in an attentional bias and heightens the perceived risk of experiencing the same event again [64]. Some even indicate a heightened perceived risk for other hazards through cross-over effects [65]. There is also evidence for generalization effects of avoidance behavior in results from mouse model studies. In mouse models of PTSD [66,67], conditioned avoidance of objects eventually turned into generalized avoidance behavior towards unknown objects. In the present study, behavioral avoidance ratings in the PTSD group were elevated. However, there was no significant association between mobility radius and self-reported avoidance behavior in the PTSD group, indicating that there might be other factors underlying spatially limited mobility in PTSD.

Another possible explanation might be drawn from research on trust in PTSD. There is evidence for an inverse relationship between trust and PTSD severity and also trust and social involvement [68]. According to these findings, a lack of interpersonal trust might explain the difficulty someone with experiences of child abuse might have in leaving a well-known area around the home, as this requires a certain amount of trust in the wider world and other people. Further research would be necessary to see whether risk perception or trust issues are underlying mechanisms of limited mobility and whether they affect some aspects of mobility (like visiting unfamiliar places) more than others (general willingness to spend time away from home).

The impairments of the PTSD group were manifold. Apart from PTSD symptoms, they had low levels of psychosocial functioning, higher psychological distress, a lower level of quality of life, lower satisfaction with social relationships and a lower health status compared to the healthy control groups. They also displayed three comorbid disorders on average. These findings are congruent with past research showing low levels of psychosocial functioning and (mental) health in individuals with experiences of child abuse [17–21,45,69]. The fact that higher depression scores and lower health scores in the PTSD group seem to account for the group difference in activity space between the PTSD group and HTC seems plausible. Depression and low physical health are generally associated with sedentary behavior and inactivity [70,71] and seem likely to limit everyday movement radius.

Several limitations of the present study must be considered. First, we only collected objective data on the participants’ mobility. Thus, no direct conclusions can be drawn about the social and emotional aspects of the participants’ behavioral patterns. Despite this, the association between movement radius and quality of life observed here indicates that limited activity space may be connected with decreased quality of life. As humans are a highly social species, staying in a restricted area could potentially lead to isolation, which is known to endanger individual well-being [72,73]. In a study of Japanese adolescents experiencing prolonged social withdrawal ("hikikomori"), participants’ social quality of life scores were lower than those of a highly depressed control group [74]. Adult survivors of child abuse often struggle to create and maintain social relationships and describe themselves as socially isolated [20,75–77]. Nevertheless, in the present study there was no significant association between movement radius and social relationships in the PTSD group. In future studies, differentiated qualitative information about participants’ whereabouts (familiarity, social contact, work relatedness, etc.) should be assessed along with information about subjective social and emotional parameters potentially associated with mobility (perceived level of mobility and avoidance, feelings of trust and anxiety, avoidance and specifically withdrawal behavior, quantity and quality of social interactions, feelings of isolation, etc.).

While the use of ambulatory assessment helped to avoid memory bias effects, control of confounding variables and adherence was limited. For future research, it would be important to assess the participants’ compliance in carrying the GPS device with them and preferably choosing a device that is even simpler to carry continuously (e.g. a wristwatch, one's personal phone) in order to increase compliance. Despite our efforts to include potential confounding variables (employment, living situation, size of hometown, health status, severity of depression) there is room for further investigation into the underlying factors of a limited activity space in patients with PTSD. Potential influencing factors worth investigating could be exercise habits or general physical activity, socioeconomic status, sleep habits and the aforementioned feelings of anxiety and social isolation. Since most previous study results are based on self-reported data, future functional impairment studies should employ a combination of subjective and objective measures to control for potential differences between self-reports and external observations.

There are some limitations regarding the representativeness of our groups. In contrast to our expectations, employment status did not vary between groups. The control groups were expected to show higher employment rates as psychosocial functioning and especially vocational functioning is generally higher in mentally healthy subjects [78]. With unemployment rates of 29–44% in the control groups, the numbers are much higher than they are generally among women in Germany (4.7% in September 2019) [79]. As a result the groups were more comparable, but it also limited the representativeness of our healthy sample and thus the external validity of our results.

The percentage of women with experiences of sexual abuse (or a combination of sexual and physical abuse) was higher in patients with PTSD than HTC (75% vs. 49%) and the mean duration of abuse was longer. There have been several studies indicating that sexual abuse has larger adverse effects than physical abuse [80] and that traumatic effects are additive [81]. In the present study, HTC and individuals with PTSD showed no difference in movement radius, despite both groups differing from HC on the weekend. These results indicate that experiences of physical and sexual violence, whether alone or in combination, may have a similar effect on activity space.

Due to restricted recruitment of only female patients for the multicenter study, only women were included into the study. Accordingly, results cannot be generalized across all genders. There are indications of a gender difference in coping strategies after experiences of child abuse or interpersonal violence in adulthood, with women showing more withdrawal and internalizing strategies than men [15,82]. It would be interesting to see in future research whether traumatized men show different mobility patterns and to assess more externalizing behavioral dimensions of interpersonal functioning, like outbursts of frustration and aggression.

## Conclusions

Despite its limitations, the present study makes an important contribution to the understanding of functional impairments in individuals with a history of child abuse. To the best of our knowledge, this is the first study that focuses on activity space in individuals with PTSD. Additionally, it employs a new yet underused methodological approach and includes a group of mentally healthy trauma control subjects. The inclusion of the HTC group can be seen as a key benefit of our study, as few studies in PTSD research have included such groups. Overall, our results indicate a potential effect of early traumatic events on activity space in later life, independent from a diagnosis of PTSD.

More research is needed to gain further insight into different areas of functional impairment after experiences of child abuse independent from a PTSD diagnosis. Results from this area could improve interventions helping individuals who suffer from long-term sequelae of abuse without meeting the full criteria of a mental disorder. Beyond that, continuously monitoring objective parameters of psychosocial functioning during the therapeutic process might serve as a useful diagnostic tool for detecting changes that go beyond mere symptom reduction in order to provide successful and holistic rehabilitation in mental health care.

## Supporting information

S1 Table Multilevel models predicting (logarithmized) time away from home: Sensitivity analyses. Table depicts unstandardized coefficients (standard errors in parentheses). Time away from home was logarithmized prior to the analyses. Results in Model 2a are the same as the results reported in Table 2 in the main document and are depicted to facilitate the comparison of the findings from the sensitivity analyses with the findings from the main analyses. Continuous predictors (hometown population, depression, health status) were centered on their sample means prior to the analyses. Number of observations = 1,563 (Model 2a), 1,517 (Model 3a), 1,416 (Model 4a); number of participants = 228 (Model 2a), 221 (Model 3a), 206 (Model 4a). *p < .05; **p < .01; ***p < .001. ^a^: weekday = 0, weekend = 1. ^b^: unemployed = 0, employed = 1. ^c^: living alone = 0, living with others = 1. ^d^: in 1,000 inhabitants.
(PDF)

S2 Table Multilevel models predicting (logarithmized) movement radius: Sensitivity analyses. Table depicts unstandardized coefficients (standard errors in parentheses). Movement radius was logarithmized prior to the analyses. Results in Model 2b are the same as the results reported in Table 3 in the main document and are depicted to facilitate the comparison of the findings from the sensitivity analyses with the findings from the main analyses. Continuous predictors (hometown population, depression, health status) were centered on their sample means prior to the analyses. Number of observations = 1,563 (Model 2b), 1,517 (Model 3b), 1,416 (Model 4b); number of participants = 228 (Model 2b), 221 (Model 3b), 206 (Model 4b). *p < .05; **p < .01; ***p < .001. ^a^: weekday = 0, weekend = 1. ^b^: unemployed = 0, employed = 1. ^c^: living alone = 0, living with others = 1. ^d^: in 1,000 inhabitants.
(PDF)
