# Supplementary material for: Life within a limited radius: Investigating activity space in women with a history of child abuse using global positioning system tracking
Source: PLoS One. 2020 May 11;15(5):e0232666. doi: 10.1371/journal.pone.0232666 (PMC7213734; doi:10.1371/journal.pone.0232666)
Supplement: S1 Table — Table depicts unstandardized coefficients (standard errors in parentheses). Time away from home was logarithmized prior to the analyses. Results in Model 2a are the same as the results reported in Table 2 in the main document and are depicted to facilitate the comparison of the findings from the sensitivity analyses with the findings from the main analyses. Continuous predictors (hometown population, depression, health status) were centered on their sample means prior to the analyses. Number of observations = 1,563 (Model 2a), 1,517 (Model 3a), 1,416 (Model 4a); number of participants = 228 (Model 2a), 221 (Model 3a), 206 (Model 4a). *p < .05; **p < .01; ***p < .001. a: weekday = 0, weekend = 1. b: unemployed = 0, employed = 1. c: living alone = 0, living with others = 1. d: in 1,000 inhabitants. (PDF) [file pone.0232666.s001.pdf]

Table S1. Multilevel models predicting (logarithmized) time away from home: Sensitivity analyses.

| | Model 2a | Model 3a | Model 4a |
|---|---|---|---|
| | Fixed effects | | |
| Intercept | 4.194*** (0.286) | 3.403*** (0.385) | 2.975*** (0.445) |
| PTSD | -0.287 (0.324) | 0.194 (0.324) | 0.823 (0.548) |
| HTC | -0.560 (0.426) | -0.496 (0.416) | -0.311 (0.428) |
| HC | (Reference) | (Reference) | (Reference) |
| Weekend[a] | -0.573* (0.285) | -0.578 (0.294) | -0.630* (0.303) |
| Employment status[b] | - | 0.705** (0.238) | 0.582* (0.251) |
| Living situation[c] | | 0.169 (0.258) | 0.253 (0.270) |
| Hometown population[d] | - | 0.0004*** (0.0001) | 0.0004*** (0.0001) |
| Depression | - | - | -.005 (.014) |
| Health status | - | - | .008 (.007) |
| PTSD x Weekend[a] | -0.421 (0.323) | -0.417 (0.333) | -0.417 (0.333) |
| HTC x Weekend[a] | -0.367 (0.424) | -0.363 (0.432) | -0.363 (0.432) |
| HC x Weekend[a] | (Reference) | (Reference) | (Reference) |
| | Random effects (variances) | | |
| Intercept | 2.872 | 2.503 | 2.488 |
| Weekend[a] | 1.307 | 1.405 | 1.441 |
| Residual (Level 1) | 3.037 | 2.973 | 2.961 |

Note. Table depicts unstandardized coefficients (standard errors in parentheses). Time away from home was logarithmized prior to the analyses. Results in Model 2a are the same as the results reported in Table 2 in the main document and are depicted to facilitate the comparison of the findings from the sensitivity analyses with the findings from the main analyses. Continuous predictors (hometown population, depression, health status) were centered on their sample means prior to the

analyses. Number of observations = 1,563 (Model 2a), 1,517 (Model 3a), 1,416 (Model 4a); number of participants = 228 (Model 2a), 221 (Model 3a), 206 (Model 4a). *$p < .05$; **$p < .01$; ***$p < .001$

[a]: weekday = 0, weekend =1.

[b]: unemployed = 0, employed =1.

[c]: living alone = 0, living with others =1.

[d]: in 1,000 inhabitants.
