# Supplementary material for: Life within a limited radius: Investigating activity space in women with a history of child abuse using global positioning system tracking
Source: PLoS One. 2020 May 11;15(5):e0232666. doi: 10.1371/journal.pone.0232666 (PMC7213734; doi:10.1371/journal.pone.0232666)
Supplement: S2 Table — Table depicts unstandardized coefficients (standard errors in parentheses). Movement radius was logarithmized prior to the analyses. Results in Model 2b are the same as the results reported in Table 3 in the main document and are depicted to facilitate the comparison of the findings from the sensitivity analyses with the findings from the main analyses. Continuous predictors (hometown population, depression, health status) were centered on their sample means prior to the analyses. Number of observations = 1,563 (Model 2b), 1,517 (Model 3b), 1,416 (Model 4b); number of participants = 228 (Model 2b), 221 (Model 3b), 206 (Model 4b). *p < .05; **p < .01; ***p < .001. a: weekday = 0, weekend = 1. b: unemployed = 0, employed = 1. c: living alone = 0, living with others = 1. d: in 1,000 inhabitants. (PDF) [file pone.0232666.s002.pdf]

Table S2. Multilevel models predicting (logarithmized) movement radius: Sensitivity analyses.

	Model 2b	Model 3b	Model 4b
	Fixed effects		
Intercept	2.002*** (0.151)	1.600*** (0.208)	1.359*** (0.249)
PTSD	-0.344* (0.171)	-0.215 (0.177)	0.245 (0.300)
HTC	-0.258 (0.225)	-0.226 (0.227)	-0.155 (0.236)
HC	(Reference)	(Reference)	(Reference)
Weekend[a]	0.186 (0.185)	0.254 (0.191)	0.205 (0.197)
Employment status[b]	-	0.403** (0.128)	0.330* (0.137)
Living situation[c]		0.145 (0.138)	0.105 (0.147)
Hometown population[d]	-	0.0000 (0.0000)	0.0000 (0.0000)
Depression	-	-	-.007 (.008)
Health status	-	-	.004 (.004)
PTSD x Weekend[a]	-0.618** (0.210)	-0.669** (0.217)	-0.628** (0.223)
HTC x Weekend[a]	-0.593* (0.276)	-0.644* (0.281)	-0.582* (0.287)
HC x Weekend[a]	(Reference)	(Reference)	(Reference)
	Random effects (variances)		
Intercept	0.726	0.676	0.699
Weekend[a]	0.612	0.630	0.644
Residual (Level 1)	1.202	1.207	1.197

Note. Table depicts unstandardized coefficients (standard errors in parentheses). Movement radius was logarithmized prior to the analyses. Results in Model 2b are the same as the results reported in Table 3 in the main document and are depicted to facilitate the comparison of the findings from the sensitivity analyses with the findings from the main analyses. Continuous predictors (hometown population, depression, health status) were centered on their sample means prior to the analyses.

Number of observations = 1,563 (Model 2b), 1,517 (Model 3b), 1,416 (Model 4b); number of participants = 228 (Model 2b), 221 (Model 3b), 206 (Model 4b). *$p < .05$; **$p < .01$; ***$p < .001$

[a]: weekday = 0, weekend =1.

[b]: unemployed = 0, employed =1.

[c]: living alone = 0, living with others =1.

[d]: in 1,000 inhabitants.
